# A survey of perceived attitudes toward psychotherapy among Thai psychiatrists

**DOI:** 10.1186/s12888-026-08303-7

**Published:** 2026-06-19

**Authors:** Warut Aunjitsakul, Kanthee Anantapong, Nisan Werachattawan, Kreuwan Jongbovonwiwat, Poom Chompoosri, Daruj Aniwattanapong, Rasmon Kalayasiri

**Affiliations:** 1 Department of Psychiatry, Faculty of Medicine, Prince of Songkla University, Hat Yai, Songkhla 90110 Thailand; 2 School of Medicine, Mae Fah Luang University, Chiang Rai, Thailand; 3 Department of Psychiatry, Faculty of Medicine, Chulalongkorn University, Bangkok, Thailand

**Keywords:** Health knowledge, Attitudes, Practice, Psychotherapy, Psychiatrists, Mental health services, Thailand, Education, Medical, Graduate

## Abstract

**Introduction:**

Psychotherapy is a crucial treatment for various mental health conditions, with its effectiveness supported by substantial evidence. This study aimed to evaluate the attitudes of psychiatrists and psychiatric trainees in Thailand toward psychotherapy, including its utilization, training preferences, barriers to access, and views on digital resources, to help guide future psychiatric training and practice in the country.

**Method:**

The 190-item ATPsySEA questionnaire evaluated attitudes toward psychotherapy across four domains: general attitudes, evidence-based utilization, barriers to access, and future training or digital perspectives. Participants were recruited through convenience sampling strategy via email and official chat channels. A total of 74 mental health professionals in Thailand completed the survey, comprising 67 psychiatrists and 7 psychiatric trainees. Data was analyzed using descriptive statistics.

**Results:**

Respondents generally held positive attitudes toward psychotherapy. No prominent differences in attitudes were observed across demographic or clinical practice characteristics. A majority (63.5%) viewed being a psychotherapist as integral to their professional identity. While time constraints were acknowledged, most (75.6%) incorporated psychotherapy into their practice, often combined with pharmacotherapy (72.9%). Cognitive Behavioral Therapy (CBT), supportive psychotherapy, and mindfulness-based psychotherapy were the most frequently utilized. Barriers to access included time constraints (45.9%), systemic limitations (24.3%), and perceived lack of skill (9.5%). Respondents expressed a strong interest in further training, particularly in CBT and basic therapeutic skills.

**Conclusion:**

Thai psychiatrists and trainees hold positive attitudes towards psychotherapy, but face barriers to its implementation such as limited clinical time. Despite these insights, the results are subject to selection and social desirability biases inherent in the recruitment method and self-reporting format, necessitating a cautious interpretation of the data.

**Trial registration:**

NA.

**Supplementary Information:**

The online version contains supplementary material available at https://doi.org/10.1186/s12888-026-08303-7.

## Introduction

Psychotherapy is recognized as a powerful therapeutic approach, applicable both as a standalone intervention and as a complement to biological treatments like medication [1, 2]. Over the past few decades, substantial scientific evidence has accumulated, underscoring its effectiveness across a range of mental health conditions [3, 4]. Emerging research in neuroscience has even illuminated the biological underpinnings of psychotherapy, with neuroimaging studies demonstrating its capacity to induce tangible changes in brain structure, volume, and metabolic activity [5–7]. Similar to pharmacological treatments, the impact of psychotherapy often correlates with the duration of intervention, with longer-term approaches showing particular promise for individuals grappling with mood instability, complex co-occurring conditions, or histories of trauma [8]. Conversely, shorter, protocol-driven psychotherapies grounded in evidence have proven effective in alleviating symptoms, enhancing interpersonal functioning, and even eliciting measurable epigenetic alterations [8–10]. Frequently, the most favorable outcomes arise from the integration of psychotherapy and pharmacotherapy [4].

While previous research across five Southeast Asian nations—Thailand, Singapore, Malaysia, Indonesia, and Vietnam—demonstrated a generally favorable attitude toward psychotherapy among both board-certified psychiatrists and trainees [11, 12], these regional findings may overlook critical intranational nuances. Specifically, a broad regional survey cannot fully account for the unique socio-cultural and systemic landscape of Thailand, where several distinct factors necessitate a more granular investigation [13].

First, Thailand’s status as a predominantly Buddhist society creates a unique psychological framework; traditional beliefs regarding karma and mindfulness often intersect with, or provide alternatives to, Western psychotherapeutic models. Second, the inequitable distribution of mental health services remains a significant systemic barrier, where low accessibility in rural areas may shape different provider attitudes compared to more urbanized neighbors like Singapore [12]. Finally, the persistent social stigma that frequently equates mental illness with ‘madness’ creates a specific clinical environment where the psychiatrist’s own perceptions are a primary gatekeeper to care [14]. Societal and systemic factors significantly shape attitudes toward psychotherapy [15]. This study moves beyond a general regional attitude to provide a critical, localized understanding of how Thai psychiatrists and trainees navigate these specific cultural and systemic hurdles. Therefore, this study aimed to evaluate the attitudes of psychiatrists and psychiatric trainees specifically within Thailand toward psychotherapy. This includes exploring its utilization in clinical practice and their preferences for future training opportunities, barriers to accessing psychotherapy and digital resources.

## Methods

This study represents a subset of a larger, multinational investigation that surveyed psychiatrists and psychiatric trainees across the Association of Southeast Asian Nations (ASEAN) countries. These countries included Indonesia, Malaysia, Singapore, Thailand, and Vietnam, all of which consented to participate, were accessible, and completed the study procedures. While data collection for the broader regional study occurred between March and August 2024, the present report is dedicated exclusively to the findings from Thailand. Although the aggregate multinational results have been published elsewhere [11, 12], this study provides a unique, intranational analysis designed to capture the specific socio-cultural nuances of the Thai mental health landscape. Consequently, this work offers a specialized focus that was not addressed in the previous cross-national publication. Participants in this study included psychiatrists within all psychiatric sub-disciplines and general psychiatry, and psychiatric trainees practicing in Thailand.

### Study population

The study population comprised psychiatrists and psychiatric trainees who were actively practicing, working, or enrolled in training programs within Thailand and possessed fluency in English. For the purposes of this research, psychiatrists were defined as medical doctors specializing in the diagnosis and treatment of mental health and substance use disorders. Psychiatric trainees referred to professionals undergoing supervised education and practical experience in the field of psychiatry. An English version of the survey instrument was utilized to ensure consistent understanding of the questionnaire items, considering the linguistic diversity prevalent in research across the ASEAN region.

### Assessment tool and instrument development

Attitudes toward psychotherapy were evaluated using the Attitudes Towards Psychotherapy in South-East Asia (ATPsySEA) questionnaire. This 190-item instrument was developed by the research team to capture a granular inventory of professional identity, treatment preferences, and systemic barriers specifically within the Southeast Asian psychiatric context. The items were generated through a multi-stage process. First, domain mapping, the domains were identified based on existing literature regarding psychotherapy in low- and middle-income countries, focusing on resource scarcity and cultural adaptations. Second, item generation, 190 items were drafted to cover four distinct areas, including general attitudes (16 items), evidence-based psychotherapy utilization across 16 diagnostic categories (80 items), barriers to access (3 items), and future training/digital perspectives (91 items). And third, content review, the items were reviewed by the co-authors (experienced psychiatrists in Thailand) to ensure relevance to local clinical practice. Responses to items were captured using a 5-point Likert scale (ranging from 1 = not at all to 5 = very much) or through multiple-choice questions, depending on the specific section. The complete questionnaire is available in the Appendix of the supplementary materials. Additionally, the tool’s breadth allows for a comprehensive descriptive map of respondent perceptions rather than a validated clinical scale, thus formal psychometric validation (e.g., factor analysis) was not performed for this exploratory study. Internal consistency was evaluated using Cronbach’s alpha for domains utilizing uniform, additive item structures: *general attitudes* (alpha = 0.88) and *future training or digital perspectives* (alpha = 0.93). For the *barriers to access* domain, the calculated alpha was expectedly low (alpha = 0.23). This low value reflects the fact that the surveyed barriers (e.g., time constraints, systemic limitations, and lack of skill) function as independent, heterogeneous, and often mutually exclusive hurdles rather than a single, unified latent construct. Conversely, a reliability coefficient could not be computed for the *evidence-based utilization* domain, as its items allowed for multiple-choice, non-additive responses per item.

In addition to the attitude assessment, socio-demographic information (e.g., country, age, gender, religion) and details about professional practice (e.g., professional status, clinical setting, years of practice, hours of clinical service) were collected.

### Data acquisition

Data were gathered using Google Forms via a convenience sampling approach. Participants were recruited through various channels, including email, social media platforms, official chat channel and advertisements. Comprehensive study information was presented on the initial page of the online form, and participants indicated their consent by selecting a designated checkbox. Eligible participants provided their socio-demographic and practice details and responded to screening questions to confirm they met the inclusion criteria: (1) being a qualified psychiatrist or a psychiatric trainee, (2) currently practicing or studying in their country of residence, and (3) possessing English language proficiency. The ATPsySEA questionnaire required approximately 35 to 40 min for completion, and participants had the option to save their progress and return to it later.

### Statistical analysis

Data are presented as frequencies and percentages. Due to the small size of the psychiatric trainee subgroup, no inferential group comparisons were performed. The R software version 4.5.2 [16] was used for data analyses.

## Results

### Socio-demographic and clinical practice characteristics

The survey included 74 respondents, the majority of whom were practicing psychiatrists (*n* = 67, 90.5%), with the remaining being psychiatric trainees (*n* = 7, 9.5%). Among the respondents, most were female (*n* = 43, 58.1%), and the most frequent age range was 31–40 years (*n* = 37, 50.0%). The predominant religious affiliation reported was Buddhism (*n* = 64, 86.5%).

Regarding clinical practice, respondents commonly worked in the following settings (listed in order of frequency): psychiatric outpatient clinics, consultation-liaison psychiatry services, psychiatric inpatient units, private practices, and emergency rooms. The most frequently reported ranges for years of clinical experience in psychiatry were 1–5 years (22, 29.7%), 6–10 years (21, 28.4%), and 11–20 years (15, 20.3%). The most common range for weekly clinical service hours reported was 21–30 h (21, 28.4%) (Table 1).

**Table 1:** Socio-demographic and clinical practice data (*N* = 74)

Variables	*n* (%)
**Gender**	
Female	43 (58.1)
Male	28 (37.8)
Non-binary	3 (4.1)
**Age range**	
20–30	12 (16.2)
>30–40	37 (50.0)
>40–50	16 (21.6)
>50–60	6 (8.1)
>60–70	3 (4.1)
**Religion**	
Buddhism	64 (86.5)
Catholicism	2 (2.7)
Christianity	1 (1.4)
Atheist or Agnostic	1 (1.4)
Non-religious	6 (8.1)
**Professional status**	
Resident/Trainee	7 (9.5)
Psychiatrist	67 (90.5)
**Clinical practice setting**	
Private practice	34 (45.9)
Psychiatric Hospital In-Patient Setting	44 (59.5)
Consultation-Liaison Psychiatry Setting	51 (68.9)
Psychiatric Out-Patient Setting	62 (83.8)
Addiction Clinic/Unit	10 (13.5)
Emergency Room	28 (37.8)
Forensic Psychiatry Setting	3 (4.1)
Child and Adolescent Psychiatry Setting	18 (24.3)
Geriatric Clinic/ Unit	7 (9.5)
**Clinical experience in psychiatry**	
1–5	22 (29.7)
6–10	21 (28.4)
11–20	15 (20.3)
21–30	6 (8.1)
31–40	4 (5.4)
Still in training	6 (8.1)
**Hours of clinical services per week**	
1–10	1 (1.4)
11–20	20 (27.0)
21–30	21 (28.4)
31–40	13 (17.6)
41–50	10 (13.5)
>50	9 (12.2)

### General attitudes towards psychotherapy

#### General attitudes

Respondents generally held positive views towards psychotherapy. A significant proportion (51.4%) rated psychotherapy as “very much” important for becoming a competent psychiatrist. Furthermore, a majority (63.5%) indicated that being a psychotherapist was “a lot” to “very much” a part of their identity as a psychiatrist or psychiatry resident.

A majority (66.2%) agreed that psychotherapy is a time-consuming approach to psychiatric problems (rated “a lot” to “very much”). Consequently, most (75.6%) reported incorporating psychotherapy into their practice at least moderately (“moderately” to “a lot”) and preferred combining it with psychopharmacology (“a lot” to “very much”; 72.9%). A considerable proportion (62.1%) reported engaging in formal psychotherapy in their clinical practice to some extent (“somewhat” to “moderately”).

Psychotherapy was viewed as highly influential, significantly impacting respondents’ clinical work (67.6% rating “a lot” or “very much”) and their personal lives (56.8% rating “a lot” or “very much”), while proving highly rewarding (58.1% rating “a lot” or “very much”). Furthermore, a strong consensus (66.2% responding “not at all” or “somewhat”) rejected the notion that psychotherapy should be conducted exclusively by psychiatrists (see Table 2).

**Table 2:** General attitudes towards psychotherapy (*N* = 74)

General attitudes towards psychotherapy	*n* (%)				
	Not at all	Somewhat	Moderately	A lot	Very much
1. How much is being a psychotherapist part of your identity as a psychiatrist/ psychiatry resident?	1 (1.4)	3 (4.1)	23 (31.1)	29 (39.2)	18 (24.3)
2. How necessary are psychotherapy skills in order to become a *competent* psychiatrist?	0 (0.0)	1 (1.4)	10 (13.5)	25 (33.8)	38 (51.4)
3. How *time consuming* is psychotherapy as a way to address psychiatric problems?	0 (0.0)	4 (5.4)	21 (28.4)	25 (33.8)	24 (32.4)
4. How much do you *incorporate* psychotherapy into your practice?	0 (0.0)	7 (9.5)	30 (40.5)	26 (35.1)	11 (14.9)
5. How much do you *integrate/combine* psychotherapy with psychopharmacology in your practice?	0 (0.0)	5 (6.8)	15 (20.3)	32 (43.2)	22 (29.7)
6. How much *formal/manualized* psychotherapy do you provide in your clinical practice?	7 (9.5)	24 (32.4)	22 (29.7)	14 (18.9)	7 (9.5)
7. How much does psychotherapy influence your *clinical work*?	1 (1.4)	2 (2.7)	21 (28.4)	33 (44.6)	17 (23.0)
8. How much does psychotherapy influence your *life outside of work*?	2 (2.7)	7 (9.5)	23 (31.1)	29 (39.2)	13 (17.6)
9. How *rewarding* is conducting psychotherapy?	1 (1.4)	9 (12.2)	21 (28.4)	32 (43.2)	11 (14.9)
10. To what extent should psychotherapy be conducted only by psychiatrists?	31 (41.9)	18 (24.3)	17 (23.0)	6 (8.1)	2 (2.7)
**Anticipated judgement by others**					
1. To what extent do you think that patients *understand* what psychotherapy is?	8 (10.8)	25 (33.8)	29 (39.2)	12 (16.2)	0 (0.0)
2. To what extent do you think that patients *know* what the effectiveness of psychotherapy is?	4 (5.4)	25 (33.8)	29 (39.2)	14 (18.9)	2 (2.7)
3. To what extent do you think patients *believe in the* effectiveness of psychotherapy?	2 (2.7)	12 (16.2)	33 (44.6)	22 (29.7)	5 (6.8)
**Personal acceptance of psychotherapy and openness to further training**					
1. How likely are you to pursue additional training in psychotherapy within your institution/program (elective or selective)?	2 (2.7)	7 (9.5)	22 (29.7)	18 (24.3)	25 (33.8)
2. How likely are you to pursue additional training in psychotherapy in addition to what your institution/program offers (e.g., workshops, seminars, courses, fellowship)?	0 (0.0)	5 (6.8)	26 (35.1)	17 (23.0)	26 (35.1)
3. How comfortable are you receiving psychotherapy education and supervision from mental health professionals, other than psychiatrists (e.g., psychologists, social workers, family physicians)?	4 (5.4)	6 (8.1)	22 (29.7)	23 (31.1)	19 (25.7)

#### Anticipated judgment by others

The majority of respondents perceived that patients had some level of understanding and awareness of the effectiveness of psychotherapy, with 72.0% rating this as “somewhat” to “moderately.” Similarly, 74.3% agreed that patients have belief in psychotherapy, rating this from “moderately” to “a lot.”

#### Personal acceptance and openness to further training

Respondents reported a strong desire to pursue additional psychotherapy training, both within their current institution or program (58.1% indicating “a lot” or “very much”) and through external workshops or courses (58.1% indicating “a lot” or “very much”). They also indicated a strong interest in receiving psychotherapy education and supervision from mental health professionals beyond psychiatrists.

### Perspectives on treatments and psychotherapies

#### Perspectives on treatments

To understand the respondents’ perspectives on evidence-based treatments and their integration, the survey explored the (1) primarily recommended and (2) given treatments, as well as (3) opinions on the most effective treatments (see Table 3 and Supplementary Tables 1 and 2). Consistent patterns emerged across these three perspectives on treatments.

**Table 3:** Commonly used evidence-based psychotherapies: What treatments are *primarily recommended* for mental disorders in your country today? (Or the treatment(s) that are in your national guidelines if guidelines exist)

Treatment options / Disorders; *n* (%)	Pharmacotherapy	Psychotherapy	Psychotherapy AND pharmacotherapy (combination)	ECT	Do not know
1. Anxiety Disorders	24 (32.4)	19 (25.7)	67 (90.5)	0 (0.0)	1 (1.4)
2. Bipolar Disorders	43 (58.1)	8 (10.8)	41 (55.4)	34 (45.9)	0 (0.0)
3. Depressive Disorders	26 (35.1)	21 (28.4)	66 (89.2)	31 (41.9)	0 (0.0)
4. Schizophrenias	51 (68.9)	8 (10.8)	34 (45.9)	40 (54.1)	0 (0.0)
5. Personality Disorders	11 (14.9)	39 (52.7)	50 (67.6)	2 (2.7)	0 (0.0)
6. Addictions	23 (31.1)	18 (24.3)	63 (85.1)	3 (4.1)	2 (2.7)
7. Eating Disorders	18 (24.3)	25 (33.8)	64 (86.5)	1 (1.4)	0 (0.0)
8. Stressor-Related Disorders (e.g., PTSD)	23 (31.1)	21 (28.4)	68 (91.9)	0 (0.0)	0 (0.0)
9. Adjustment Disorders	15 (20.3)	47 (63.5)	43 (58.1)	0 (0.0)	0 (0.0)
10. Conduct and Impulse Control Disorders	26 (35.1)	19 (25.7)	55 (74.3)	1 (1.4)	4 (5.4)
11. Neuro-developmental Disorders	43 (58.1)	12 (16.2)	39 (52.7)	3 (4.1)	5 (6.8)
12. Cognitive Disorders	41 (55.4)	14 (18.9)	40 (54.1)	4 (5.4)	3 (4.1)
13. Obsessive-Compulsive Disorders	22 (29.7)	18 (24.3)	68 (91.9)	6 (8.1)	0 (0.0)
14. Sleep Disorders	28 (37.8)	18 (24.3)	60 (81.1)	0 (0.0)	1 (1.4)
15. Sexual Disorders	21 (28.4)	26 (35.1)	49 (66.2)	1 (1.4)	6 (8.1)
16. Somatic Symptom Disorders	16 (21.6)	24 (32.4)	58 (78.4)	0 (0.0)	3 (4.1)

ECT: Electro-Convulsive Therapy; PTSD: Post-Traumatic Stress Disorder

Pharmacotherapy was the agreed-upon primary treatment for individuals with schizophrenia, bipolar disorders, neurodevelopmental disorders, and cognitive disorders. Conversely, psychotherapy was the primary treatment recommended and provided for adjustment disorder, personality disorders, and sexual disorders. Combination therapy, involving both psychotherapy and pharmacotherapy, was commonly prescribed for individuals with stressor-related disorders, obsessive-compulsive disorders (OCD), addictions, anxiety disorders, depressive disorders, and eating disorders. Regarding Electroconvulsive Therapy (ECT), respondents consistently agreed that it was an appropriate treatment for individuals with schizophrenia, bipolar disorders, and depressive disorders, in that order of agreement across the three aspects.

#### Perspectives on psychotherapies

To gain insight into the respondents’ perspectives on psychotherapies, the survey inquired about respondent impressions of recommended psychotherapies and their opinions on the most effective approaches (see Table 4 and Supplementary Table 3). Analysis of both tables reveals a general trend in psychotherapy use, with Cognitive Behavioral Therapy (CBT) being the most frequently cited approach, followed by supportive psychotherapy and mindfulness-based therapy.

**Table 4:** Commonly used evidence-based psychotherapies: Which of the following psychotherapies are *recommended* in your country as treatment options for mental disorders? You can choose as many as applicable

Therapies/Disorders; *n* (%)	CBT	Psychodynamic	Mindfulness	Motivational Interviewing	DBT	ACT	Supportive	Interpersonal
1. Anxiety Disorders	70 (94.6)	19 (25.7)	50 (67.6)	2 (2.7)	3 (4.1)	12 (16.2)	42 (56.8)	12 (16.2)
2. Bipolar Disorders	42 (56.8)	8 (10.8)	18 (24.3)	3 (4.1)	6 (8.1)	11 (14.9)	48 (64.9)	20 (27.0)
3. Depressive Disorders	69 (93.2)	21 (28.4)	53 (71.6)	3 (4.1)	14 (18.9)	26 (35.1)	53 (71.6)	40 (54.1)
4. Schizophrenias	31 (41.9)	7 (9.5)	6 (8.1)	2 (2.7)	0 (0.0)	8 (10.8)	53 (71.6)	9 (12.2)
5. Personality Disorders	40 (54.1)	39 (52.7)	34 (45.9)	5 (6.8)	59 (79.7)	26 (35.1)	50 (67.6)	31 (41.9)
6. Addictions	43 (58.1)	18 (24.3)	10 (13.5)	68 (91.9)	9 (12.2)	15 (20.3)	35 (47.3)	9 (12.2)
7. Eating Disorders	59 (79.7)	25 (33.8)	17 (23.0)	12 (16.2)	5 (6.8)	10 (13.5)	34 (45.9)	11 (14.9)
8. Stressor-Related Disorders (e.g., PTSD)	58 (78.4)	21 (28.4)	33 (44.6)	3 (4.1)	8 (10.8)	12 (16.2)	44 (59.5)	7 (9.5)
9. Adjustment Disorders	47 (63.5)	47 (63.5)	34 (45.9)	4 (5.4)	6 (8.1)	13 (17.6)	35 (47.3)	9 (12.2)
10. Conduct and Impulse Control Disorders	39 (52.7)	19 (25.7)	19 (25.7)	8 (10.8)	5 (6.8)	6 (8.1)	43 (58.1)	10 (13.5)
11. Neuro-developmental Disorders	25 (33.8)	12 (16.2)	9 (12.2)	2 (2.7)	2 (2.7)	7 (9.5)	51 (68.9)	12 (16.2)
12. Cognitive Disorders	23 (31.1)	14 (18.9)	10 (13.5)	0 (0.0)	4 (5.4)	7 (9.5)	52 (70.3)	5 (6.8)
13. Obsessive-Compulsive Disorders	67 (90.5)	18 (24.3)	20 (27.0)	2 (2.7)	3 (4.1)	7 (9.5)	29 (39.2)	5 (6.8)
14. Sleep Disorders	66 (89.2)	18 (24.3)	16 (21.6)	1 (1.4)	5 (6.8)	4 (5.4)	30 (40.5)	5 (6.8)
15. Sexual Disorders	51 (68.9)	26 (35.1)	9 (12.2)	2 (2.7)	3 (4.1)	5 (6.8)	39 (52.7)	8 (10.8)
16. Somatic Symptom Disorders	54 (73.0)	24 (32.4)	22 (29.7)	1 (1.4)	3 (4.1)	9 (12.2)	44 (59.5)	7 (9.5)

ACT: Acceptance and Commitment Therapy; CBT: Cognitive Behavioural Therapy; DBT: Dialectical Behaviour Therapy; ECT: Electro-Convulsive Therapy; PTSD: Post-Traumatic Stress Disorder

We observed some consistency between the recommended and perceived effective psychotherapies. CBT was commonly indicated for individuals with anxiety disorders, depressive disorders, OCD, and eating disorders. Supportive psychotherapy was frequently mentioned for depressive disorders, schizophrenia spectrum disorders, bipolar disorders, neurodevelopmental disorders, and cognitive disorders. Mindfulness-based therapy was associated with anxiety disorders, depressive disorders, personality disorders, and adjustment disorder.

The psychotherapies most prominently reported by respondents for specific disorders included: psychodynamic psychotherapy for adjustment disorder, personality disorder, and sexual disorders; motivational interviewing for addictions; dialectical behavior therapy (DBT) for personality disorder; acceptance and commitment therapy (ACT) for depressive disorder and personality disorder; and interpersonal therapy for depressive disorder and personality disorder.

### Barriers to accessing psychotherapy

The three most common incentives cited for integrating psychotherapy into clinical practice were treatment capacity (47.3%), economic factors (20.3%), and clinical effectiveness (14.9%). Conversely, the main barriers to integrating psychotherapy were perceived to be the time-consuming nature of psychotherapy (45.9%), limitations within the mental health system (24.3%), and a perceived lack of skill in psychotherapy among professionals or patients (9.5%).

These findings aligned with the challenges reported by healthcare professionals when providing psychotherapy. The most significant challenges were the time-consuming nature of psychotherapy (40.5%), work overload (24.3%), and limited skills in conducting psychotherapy (10.8%) (Supplementary Table 4).

### Future perspectives on psychotherapy

#### Expected changes in training

For recommendations regarding psychotherapy training, respondents rated CBT, supportive psychotherapy, motivational interviewing, mindfulness-based therapy, and systems/family systems therapy as very important. Psychoanalysis was the least preferred for training, while familiarity with feminist therapy, person-centered therapy, and mentalization-based therapy was low.

For further training in therapeutic methods, relaxation techniques, communication skills, problem-solving techniques, cognitive restructuring, and emotional regulation were highly valued. Free association, hypnosis, aversive conditioning, dreamwork, and the use of psychedelic drugs were considered unimportant. Awareness of bibliotherapy, psychedelic drugs, cathartic methods, paradoxical interventions, and contingency management was limited.

Regarding expectations for other professionals providing psychotherapy, psychologists, psychiatrists, and master-level family therapists were commonly supported, in that order. Videoconferencing, flexible platforms, and smartphone apps were the preferred delivery methods. Additionally, respondents strongly agreed on the importance of individual therapy, crisis intervention, and couple/marital therapy, suggesting a need for changes in their structural implementation (See Table 5 and Supplementary Table 5).

**Table 5:** Expected changes in psychotherapy training in Thailand: psychotherapies and therapeutic methods

Expected changes in psychotherapy training	*n* (%)					
Psychotherapies ^a^	Not at all	Somewhat	Moderately	A lot	Very much	Not at all
1. Mindfulness Therapies	1 (1.4)	5 (6.8)	15 (20.3)	17 (23.0)	33 (44.6)	3 (4.1)
2. Cognitive-Behavior Therapy	0 (0.0)	1 (1.4)	6 (8.1)	15 (20.3)	46 (62.2)	6 (8.1)
3. Motivational Interviewing	0 (0.0)	4 (5.4)	10 (13.5)	13 (17.6)	42 (56.8)	5 (6.8)
4. Acceptance & Commitment Therapy	2 (2.7)	7 (9.5)	21 (28.4)	21 (28.4)	15 (20.3)	8 (10.8)
5. Dialectical Behavior Therapy	3 (4.1)	9 (12.2)	19 (25.7)	24 (32.4)	13 (17.6)	6 (8.1)
6. Exposure Therapies	0 (0.0)	3 (4.1)	16 (21.6)	24 (32.4)	23 (31.1)	8 (10.8)
7. Interpersonal Therapy	0 (0.0)	5 (6.8)	20 (27.0)	25 (33.8)	17 (23.0)	7 (9.5)
8. Systems/Family Systems Therapy	0 (0.0)	3 (4.1)	13 (17.6)	23 (31.1)	30 (40.5)	5 (6.8)
9. Relational Therapy	2 (2.7)	7 (9.5)	22 (29.7)	18 (24.3)	14 (18.9)	11 (14.9)
10. Supportive Therapy	1 (1.4)	0 (0.0)	8 (10.8)	12 (16.2)	45 (60.8)	8 (10.8)
11. Feminist Therapy	5 (6.8)	12 (16.2)	21 (28.4)	10 (13.5)	8 (10.8)	18 (24.3)
12. Narrative Therapy	4 (5.4)	10 (13.5)	28 (37.8)	13 (17.6)	10 (13.5)	9 (12.2)
13. Mentalization-Based Therapy	3 (4.1)	9 (12.2)	24 (32.4)	12 (16.2)	12 (16.2)	14 (18.9)
14. EMDR Therapy	1 (1.4)	4 (5.4)	23 (31.1)	26 (35.1)	15 (20.3)	5 (6.8)
15. Person Centered Therapy	6 (8.1)	9 (12.2)	14 (18.9)	20 (27.0)	7 (9.5)	18 (24.3)
16. Psychodynamic Therapy	4 (5.4)	8 (10.8)	18 (24.3)	15 (20.3)	23 (31.1)	6 (8.1)
17. Play Therapy	4 (5.4)	12 (16.2)	23 (31.1)	16 (21.6)	12 (16.2)	7 (9.5)
18. Humanistic Therapy	4 (5.4)	10 (13.5)	23 (31.1)	18 (24.3)	8 (10.8)	11 (14.9)
19. Psychoanalysis (classical)	18 (24.3)	17 (23.0)	16 (21.6)	11 (14.9)	7 (9.5)	5 (6.8)
**Therapeutic methods** ^**b**^						
1. Online self-help	6 (8.1)	8 (10.8)	15 (20.3)	20 (27.0)	23 (31.1)	2 (2.7)
2. Virtual reality methods	6 (8.1)	13 (17.6)	21 (28.4)	18 (24.3)	13 (17.6)	3 (4.1)
3. Routine outcome monitoring	1 (1.4)	8 (10.8)	17 (23.0)	21 (28.4)	22 (29.7)	5 (6.8)
4. Teaching emotional regulation	0 (0.0)	2 (2.7)	6 (8.1)	15 (20.3)	44 (59.5)	7 (9.5)
5. Fostering the therapeutic alliance	1 (1.4)	2 (2.7)	14 (18.9)	18 (24.3)	34 (45.9)	5 (6.8)
6. Acceptance methods	1 (1.4)	4 (5.4)	10 (13.5)	27 (36.5)	27 (36.5)	5 (6.8)
7. Homework assignments	0 (0.0)	4 (5.4)	23 (31.1)	19 (25.7)	22 (29.7)	6 (8.1)
8. Relapse prevention	0 (0.0)	1 (1.4)	11 (14.9)	16 (21.6)	40 (54.1)	6 (8.1)
9. Social networking interventions	0 (0.0)	3 (4.1)	16 (21.6)	27 (36.5)	25 (33.8)	3 (4.1)
10. Meditation methods	0 (0.0)	5 (6.8)	19 (25.7)	24 (32.4)	21 (28.4)	5 (6.8)
11. Behavioral activation	0 (0.0)	2 (2.7)	7 (9.5)	19 (25.7)	39 (52.7)	7 (9.5)
12. Cognitive restructuring	0 (0.0)	1 (1.4)	7 (9.5)	14 (18.9)	45 (60.8)	7 (9.5)
13. Behavioral rehearsal/in-session roleplaying	2 (2.7)	4 (5.4)	15 (20.3)	24 (32.4)	23 (31.1)	6 (8.1)
14. Recommendation of self-help	1 (1.4)	4 (5.4)	11 (14.9)	27 (36.5)	27 (36.5)	4 (5.4)
15. Problem-solving techniques	0 (0.0)	1 (1.4)	8 (10.8)	12 (16.2)	46 (62.2)	7 (9.5)
16. Communication skills	0 (0.0)	1 (1.4)	4 (5.4)	13 (17.6)	48 (64.9)	8 (10.8)
17. Interpersonal support	0 (0.0)	1 (1.4)	11 (14.9)	14 (18.9)	41 (55.4)	7 (9.5)
18. Mentalization	0 (0.0)	4 (5.4)	15 (20.3)	19 (25.7)	27 (36.5)	9 (12.2)
19. Forgiveness methods	1 (1.4)	4 (5.4)	16 (21.6)	23 (31.1)	22 (29.7)	8 (10.8)
20. Self-control methods	1 (1.4)	4 (5.4)	9 (12.2)	17 (23.0)	37 (50.0)	6 (8.1)
21. Expressing caring & warmth	0 (0.0)	3 (4.1)	9 (12.2)	16 (21.6)	41 (55.4)	5 (6.8)
22. Relaxation techniques	0 (0.0)	2 (2.7)	7 (9.5)	9 (12.2)	49 (66.2)	7 (9.5)
23. In vivo exposure	0 (0.0)	4 (5.4)	19 (25.7)	25 (33.8)	20 (27.0)	6 (8.1)
24. Therapeutic journaling	2 (2.7)	7 (9.5)	19 (25.7)	22 (29.7)	15 (20.3)	9 (12.2)
25. Solution-focused methods	1 (1.4)	2 (2.7)	11 (14.9)	23 (31.1)	32 (43.2)	5 (6.8)
26. Advice/direct guidance	1 (1.4)	4 (5.4)	12 (16.2)	15 (20.3)	37 (50.0)	5 (6.8)
27. Bibliotherapy	4 (5.4)	7 (9.5)	22 (29.7)	16 (21.6)	7 (9.5)	18 (24.3)
28. Psychedelic drugs	12 (16.2)	9 (12.2)	24 (32.4)	6 (8.1)	7 (9.5)	16 (21.6)
29. Therapist self-disclosure	1 (1.4)	15 (20.3)	22 (29.7)	13 (17.6)	14 (18.9)	9 (12.2)
30. Imagery	0 (0.0)	9 (12.2)	28 (37.8)	19 (25.7)	11 (14.9)	7 (9.5)
31. Contingency management methods	1 (1.4)	6 (8.1)	26 (35.1)	15 (20.3)	13 (17.6)	13 (17.6)
32. Metaphors	0 (0.0)	4 (5.4)	16 (21.6)	21 (28.4)	30 (40.5)	3 (4.1)
33. Challenges/confrontation	1 (1.4)	8 (10.8)	18 (24.3)	24 (32.4)	23 (31.1)	0 (0.0)
34. Biofeedback	2 (2.7)	5 (6.8)	30 (40.5)	17 (23.0)	16 (21.6)	4 (5.4)
35. Systematic desensitization	0 (0.0)	3 (4.1)	16 (21.6)	26 (35.1)	25 (33.8)	4 (5.4)
36. Analysis of resistance	2 (2.7)	9 (12.2)	21 (28.4)	18 (24.3)	17 (23.0)	7 (9.5)
37. Hypnosis	24 (32.4)	17 (23.0)	17 (23.0)	6 (8.1)	6 (8.1)	4 (5.4)
38. Transference interpretation	5 (6.8)	17 (23.0)	14 (18.9)	16 (21.6)	16 (21.6)	6 (8.1)
39. Paradoxical interventions	6 (8.1)	20 (27.0)	16 (21.6)	11 (14.9)	6 (8.1)	15 (20.3)
40. Cathartic methods	11 (14.9)	16 (21.6)	14 (18.9)	8 (10.8)	9 (12.2)	16 (21.6)
41. Dream work	13 (17.6)	25 (33.8)	16 (21.6)	9 (12.2)	4 (5.4)	7 (9.5)
42. Aversive conditioning	15 (20.3)	20 (27.0)	14 (18.9)	8 (10.8)	9 (12.2)	8 (10.8)
43. Free association	25 (33.8)	13 (17.6)	16 (21.6)	13 (17.6)	3 (4.1)	4 (5.4)

Note:

^a^ How likely are you to *advice training* in following *psychotherapies* as a part of future clinical work (for both residents and psychiatrists)?

^b^ How likely are you to suggest additional *training* in following *therapeutic methods* as a part of future clinical work (for both residents and psychiatrists

#### Internet-based psychotherapy

Regarding innovative methods, most respondents were less familiar with online therapy and web-based treatment. The most common challenges identified for internet-based psychotherapy were concerns about clinical effectiveness (44.6%), lack of support from the healthcare system/health insurance (33.8%), and issues related to accessibility and cost of access (32.4%) (Supplementary Tables 6 and 7).

## Discussion

This survey mapped the attitudes toward psychotherapy among 67 Thai psychiatrists and 7 psychiatric trainees. Most respondents possessed 1–10 years of clinical experience (58.1%) and provided 21–30 h of weekly clinical service (28.4%). While these findings offer valuable insights into local baseline perspectives, they must be interpreted cautiously due to selection and social desirability biases inherent in our convenience sampling and self-reporting methodology.

### General attitudes towards psychotherapy

Both psychiatrists and psychiatric trainees expressed positive attitudes towards psychotherapy and favored combining it with psychopharmacology, consistent with Western countries [17–19], reflecting the preference of psychotherapeutic practice which are viewed favorably across diverse cultural contexts. Although there was a positive trend towards psychotherapy, the proliferation of psychopharmacology might lead to a decrease in psychotherapy specialization [20]. While acknowledging that psychotherapy is time-consuming and can be challenging to implement formally in clinical settings, respondents were open to other mental health professionals providing psychotherapy, thereby extending its reach beyond psychiatrists. Furthermore, they showed a continued interest in pursuing psychotherapy training both within and outside their organizations. These findings align with the documented challenges of limited mental health resources in low- to middle-income countries, including Thailand, where cultural factors and socioeconomic disparities can further exacerbate the disproportionate ratio of doctors to patients and access to care [21, 22]. Consequently, the heavy workload often prevents psychiatrists from providing psychotherapy, potentially leading to a preference for prescribing medication [21, 22]. Expanding the provision of psychotherapy to other trained mental health professionals could help alleviate this burden and strengthen the overall healthcare system [23, 24].

Our results reported high baseline valuation of psychotherapy mirrors international data across several low- and middle-income nations, where early career psychiatrists consistently view psychotherapy as core to their professional identity. For instance, in countries like Nepal, Iran, Turkey, and Brazil, trainees and early career clinicians overwhelmingly voice a strong desire for expanded psychotherapy training [25–28]. Pan-European surveys have similarly confirmed this trend, demonstrating a powerful motivation for psychotherapy training among European psychiatrists despite persistent structural challenges, such as funding deficits, a scarcity of qualified supervisors, and difficulties in securing dedicated time away from primary clinical duties [29, 30].

However, structural challenges in translating these attitudes into practice vary globally. In Nepal, clinical interest is heavily bottlenecked by a severe lack of institutional access to formal training [25]. Conversely, in countries where training is nationally mandated, such as Iran and Brazil, different systemic hurdles emerge; for example, the high financial burden of securing high-quality supervision and advanced practical training limits real-world application [27, 28]. These global hurdles parallel the time constraints and institutional resource limitations reported by our Thai survey.

Furthermore, the preference among Thai respondents for structured, short-term modalities like CBT over classical psychoanalysis aligns with contemporary international curricular shifts. In Turkey and Brazil, psychiatry training has increasingly pivoted away from unstructured psychodynamic models toward protocol-driven, evidence-based frameworks to maximize treatment capacity within restricted clinical hours [26, 27]. Collectively, these cross-national comparisons demonstrate that while positive attitudes toward psychotherapy are globally universal, robust local curricular standardization and financial support are essential to bridge the gap between clinical interest and actual practice.

While both Thai psychiatrists and trainees maintain highly positive baseline attitudes toward psychotherapy, translating this preference into clinical practice is heavily mediated by systemic factors. Similar tensions are well-documented in other Asian Low- and Middle-Income Countries, such as Iran, Pakistan, where despite favorable clinician attitudes and a recognized demand for psychological interventions, actual delivery is profoundly hindered by a shortage of trained professionals, heavy clinical workloads, and a lack of structured institutional support [31–33]. Conversely, in many European countries, while structural resources and multi-disciplinary mental health teams are often more robustly established, psychiatrists still face a parallel shift: an escalating pressure to prioritize psychopharmacology and shorter diagnostic consultations over formal, long-term psychotherapy due to high service demands and evolving insurance reimbursement models [34].

### Perspectives on treatments and psychotherapies

Psychotherapy and pharmacotherapy were frequently reported as common treatments across a range of mental disorders, particularly stressor-related disorders, OCD, addictions, anxiety disorders, depressive disorders, and eating disorders. Among psychotherapies, CBT, supportive psychotherapy, and mindfulness therapy were the most preferred approaches, which were similar to other SEA countries [11]. Current findings show distinct preferences for certain therapies, such as psychodynamic approaches for personality, sexual, and adjustment disorders (other preferences see in the result subsection: Perspective on Psychotherapies). However, the potential for modularized, person-centered intervention combinations remains largely unexplored [35]. This highlights a critical gap in the literature: while real-world practice routinely relies on combination treatments (including concurrent psychotherapy and pharmacotherapy), these combinations rarely follow systematically evaluated or empirically validated protocols [35, 36].

### Barriers to accessing psychotherapy

The primary incentives identified for integrating psychotherapy were treatment capacity, economic factors, and perceived clinical effectiveness. These findings can be understood within the context of: (1) the mental health system in Thailand, which faces a shortage of mental health providers and limited financial resources to adequately cover treatment, particularly psychotherapy [21, 22, 37]; and (2) a potential feeling of professional inadequacy in providing psychotherapy, which may deter its integration [38]. Furthermore, the perceived scarcity of robust evidence-based outcomes for some psychotherapies might contribute to hesitancy in their application in real-world practice, despite their potential clinical utility [39, 40].

Consistently reported barriers and challenges related to accessing psychotherapy included its time-consuming nature, practitioner work overload, limitations within the mental health system, and a perceived lack of skill in psychotherapy among both professionals and patients. This perception reflects the concern for additional support for mental health professionals, particularly psychiatrists who constituted the majority of respondents. Such support should extend beyond simply increasing the number of trained psychiatrists to include other mental healthcare professionals [41, 42]. Moreover, regular workshops and updates on psychotherapy knowledge and techniques are preferred to enhance psychotherapeutic skills. Providing supervision for early-career psychiatrists could also provide benefit for ensuring their well-being and guiding their therapeutic practice, as supported by organizations such as the Network of Early Career Psychiatrist of Thailand (NECpT) with the Psychiatric Association of Thailand [43].

### Future perspectives on psychotherapy

Respondents indicated a strong interest in training across various psychotherapies, including CBT, supportive psychotherapy, motivational interviewing, mindfulness-based therapy, and systems/family systems therapy. Notably, basic therapeutic skills and techniques, such as relaxation, communication, problem-solving, cognitive restructuring, and emotional regulation, were highlighted as crucial areas for future training. Strengthening these fundamental skills is likely seen as essential for improving competency in integrating psychotherapy, perhaps even more than simply acquiring knowledge of diverse psychotherapeutic schools [44]. Psychoanalysis and its associated methods (e.g., free association, hypnosis, dreamwork) generated the least interest, potentially due to perceived limited utility and effectiveness in clinical practice.

To enhance access to psychological interventions, respondents supported increasing the number of psychotherapists, including psychologists, psychiatrists, and master-level family therapists. While open to utilizing videoconferencing, flexible platforms, and smartphone apps for therapy delivery, they expressed unfamiliarity with internet-based approaches and raised concerns regarding clinical effectiveness, lack of healthcare system/insurance support, and accessibility/cost issues. Addressing these concerns through adequate staff support for innovative methods, improved insurance coverage for both pharmacotherapy and psychotherapy [37], and enhanced referral systems (including better doctor-to-doctor and doctor-to-patient connections) could facilitate the adoption of telepsychotherapy [45, 46].

Furthermore, respondents suggested restructuring therapy formats, particularly individual therapy, crisis intervention, and couple/marital therapy. Addressing these issues could be helpful for these modern days such as burnout, mental capacity limitations, and relationship conflicts prevalent in current and future generations [47–49]. They emphasized the need for concise, targeted, practical, and easily applicable therapy structures [50].

The strong preference among Thai respondents for structured training in core competencies (such as CBT, supportive therapy, and motivational interviewing) and basic therapeutic skills and techniques (such as relaxation, problem-solving, cognitive restructuring, and emotional regulation) over traditional modalities like classical psychoanalysis aligns closely with contemporary international training trends. In European psychiatric residency programs, there has been a steady, decades-long curricular shift away from unstructured psychoanalytic models toward short-term, evidence-based, protocol-driven modalities like CBT [51]. Moreover, to maximize treatment capacity within their current systemic constraints, Thai practitioners expressed a clear preference for more personalized, concise, targeted, and easily applicable therapeutic structures. Implementing these practical, streamlined intervention formats is highly suitable for addressing both current and evolving future mental health challenges [35, 52].

### Strengths and limitations

This survey of psychiatrists and psychiatric trainees in Thailand offers deeper understanding into their attitudes towards psychotherapy. The study’s exploration of general attitudes, perspectives on treatments and psychotherapies, barriers to access, and future directions provides an extensive overview. Furthermore, the identified preferences for psychotherapy training and delivery methods offer practical need for curriculum development and service planning.

Several limitations must be acknowledged. First, the ATPsySEA questionnaire is an unvalidated instrument; therefore, its reliability and reproducibility remain unconfirmed. However, the questionnaire extensively addressed the study’s primary objectives aiming to explore utilization of psychotherapy in clinical practice and their preferences for future training opportunities, barriers to accessing psychotherapy and digital resources. Second, the use of convenience sampling via social media and email likely introduced selection and volunteer bias, potentially over-representing psychiatrists with a pre-existing interest in psychotherapy or English-language academic discourse. Third, as we did not track the total number of individuals who viewed the recruitment advertisements, we are unable to report a formal response rate or analyze non-response bias. The reported 90.5% figure reflects only the composition of the final sample, not a recruitment success rate. Fourth, the small number of trainees (*n* = 7) precludes meaningful statistical comparisons between residents and senior psychiatrists. Finally, the quantitative nature of the study did not allow for a deep exploration, suggesting a need for qualitative research.

### Implications for practice, education, and research

The preference for integrated treatment suggests several potential avenues for future development. Formulating guidelines and enhancing mental health literacy within the country could help bridge the gap between pharmacotherapy and psychological interventions. Additionally, exploring ways to improve clinical accessibility [21] and insurance coverage [37] may address existing structural barriers. Supporting professionals in skill development [50] and establishing telepsychotherapy through improved infrastructure and staff training [39, 46] could further enhance service delivery. For education, there may be value in prioritizing core competencies—such as CBT, mindfulness, and emotional regulation—while broadening exposure to less familiar or emerging modalities. Future research should continue to evaluate culturally adapted training models, telepsychotherapy in the Thai context, and the qualitative reasons behind provider and patient attitudes.

## Conclusion

This survey indicates a generally positive attitude toward psychotherapy among Thai psychiatrists and trainees, alongside a recognition of its clinical value. The expressed interest in further training and digital delivery suggests an opportunity to address existing barriers and refine how psychotherapeutic interventions are integrated into the Thai mental healthcare system. Future initiatives centered on educational support, structural considerations, and targeted research may help in realizing the potential for more accessible, integrated care.

## Supplementary Information

Below is the link to the electronic supplementary material.

Supplementary Material 1

Supplementary Material 2

Supplementary Material 3

Supplementary Material 4

Supplementary Material 5

Supplementary Material 6

Supplementary Material 7

## Data Availability

The data that support the findings of this study are not openly available due to reasons of sensitivity and are available from the corresponding author upon reasonable request.

## Abbreviations

ACT: Acceptance and commitment therapy
ASEAN: The Association of Southeast Asian Nations
ATPsySEA: The Attitudes Towards Psychotherapy in South-East Asia
CBT: Cognitive Behavioral Therapy
DBT: Dialectical behavior therapy
ECT: Electroconvulsive Therapy
NECpT: The Network of Early Career Psychiatrist of Thailand
OCD: obsessive-compulsive disorder
