# Supplementary material for: A survey of perceived attitudes toward psychotherapy among Thai psychiatrists
Source: BMC Psychiatry. 2026 Jun 19;26:692. doi: 10.1186/s12888-026-08303-7 (PMC13540908; doi:10.1186/s12888-026-08303-7)
Supplement: Supplementary file 1 [file 12888_2026_8303_MOESM1_ESM.docx]

**Supplementary Table 1** Commonly used evidence-based psychotherapies: What treatments are *primarily given* for mental disorders in your country today? (The treatments you believe a patient is most likely to receive when seeking help for the following disorders).

| **Treatment options / Disorders; n (%)** | **Pharmacotherapy** | **Psychotherapy** | **Psychotherapy AND pharmacotherapy (combination)** | **ECT** | **Do not know** |
| --- | --- | --- | --- | --- | --- |
| 1. Anxiety Disorders | 46 (62.2) | 16 (21.6) | 41 (55.4) | 0 (0.0) | 0 (0.0) |
| 2. Bipolar Disorders | 59 (79.7) | 7 (9.5) | 26 (35.1) | 15 (20.3) | 0 (0.0) |
| 3. Depressive Disorders | 41 (55.4) | 18 (24.3) | 50 (67.6) | 14 (18.9) | 1 (1.4) |
| 4. Schizophrenias | 64 (86.5) | 5 (6.8) | 18 (24.3) | 21 (28.4) | 0 (0.0) |
| 5. Personality Disorders | 36 (48.6) | 26 (35.1) | 41 (55.4) | 1 (1.4) | 2 (2.7) |
| 6. Addictions | 40 (54.1) | 13 (17.6) | 42 (56.8) | 1 (1.4) | 4 (5.4) |
| 7. Eating Disorders | 34 (45.9) | 16 (21.6) | 48 (64.9) | 1 (1.4) | 2 (2.7) |
| 8. Stressor-Related Disorders (e.g., PTSD) | 35 (47.3) | 12 (16.2) | 49 (66.2) | 1 (1.4) | 1 (1.4) |
| 9. Adjustment Disorders | 32 (43.2) | 31 (41.9) | 39 (52.7) | 0 (0.0) | 0 (0.0) |
| 10. Conduct and Impulse Control Disorders | 41 (55.4) | 12 (16.2) | 41 (55.4) | 1 (1.4) | 2 (2.7) |
| 11. Neuro-developmental Disorders | 48 (64.9) | 11 (14.9) | 31 (41.9) | 1 (1.4) | 3 (4.1) |
| 12. Cognitive Disorders | 55 (74.3) | 9 (12.2) | 24 (32.4) | 1 (1.4) | 2 (2.7) |
| 13. Obsessive-Compulsive Disorders | 43 (58.1) | 12 (16.2) | 44 (59.5) | 4 (5.4) | 0 (0.0) |
| 14. Sleep Disorders | 49 (66.2) | 10 (13.5) | 34 (45.9) | 0 (0.0) | 1 (1.4) |
| 15. Sexual Disorders | 36 (48.6) | 40 (54.1) | 14 (18.9) | 0 (0.0) | 6 (8.1) |
| 16. Somatic Symptom Disorders | 40 (54.1) | 17 (23.0) | 42 (56.8) | 0 (0.0) | 0 (0.0) |

ECT: Electro-Convulsive Therapy; PTSD: Post-Traumatic Stress Disorder
