# Supplementary material for: A survey of perceived attitudes toward psychotherapy among Thai psychiatrists
Source: BMC Psychiatry. 2026 Jun 19;26:692. doi: 10.1186/s12888-026-08303-7 (PMC13540908; doi:10.1186/s12888-026-08303-7)
Supplement: Supplementary file 3 [file 12888_2026_8303_MOESM3_ESM.docx]

**Supplementary Table 3** Commonly used evidence-based psychotherapies: In your opinion which of these psychotherapies are effective in the treatment of mental disorders? You can choose as many as applicable.

| **Therapies/Disorders; n (%)** | **CBT** | **Psychodynamic** | **Mindfulness** | **Motivational Interviewing** | **DBT** | **ACT** | **Supportive** | **Interpersonal** |
| --- | --- | --- | --- | --- | --- | --- | --- | --- |
| 1. Anxiety Disorders | 70 (94.6) | 16 (21.6) | 49 (66.2) | 4 (5.4) | 6 (8.1) | 22 (29.7) | 42 (56.8) | 16 (21.6) |
| 2. Bipolar Disorders | 49 (66.2) | 7 (9.5) | 23 (31.1) | 5 (6.8) | 5 (6.8) | 13 (17.6) | 54 (73.0) | 25 (33.8) |
| 3. Depressive Disorders | 70 (94.6) | 18 (24.3) | 46 (62.2) | 6 (8.1) | 17 (23.0) | 30 (40.5) | 52 (70.3) | 37 (50.0) |
| 4. Schizophrenias | 29 (39.2) | 9 (12.2) | 18 (24.3) | 4 (5.4) | 2 (2.7) | 11 (14.9) | 56 (75.7) | 15 (20.3) |
| 5. Personality Disorders | 40 (54.1) | 26 (35.1) | 36 (48.6) | 6 (8.1) | 55 (74.3) | 28 (37.8) | 48 (64.9) | 43 (58.1) |
| 6. Addictions | 46 (62.2) | 13 (17.6) | 20 (27.0) | 63 (85.1) | 14 (18.9) | 18 (24.3) | 30 (40.5) | 10 (13.5) |
| 7. Eating Disorders | 62 (83.8) | 16 (21.6) | 24 (32.4) | 15 (20.3) | 9 (12.2) | 10 (13.5) | 31 (41.9) | 13 (17.6) |
| 8. Stressor-Related Disorders (e.g., PTSD) | 56 (75.7) | 12 (16.2) | 41 (55.4) | 3 (4.1) | 7 (9.5) | 11 (14.9) | 43 (58.1) | 11 (14.9) |
| 9. Adjustment Disorders | 50 (67.6) | 31 (41.9) | 36 (48.6) | 7 (9.5) | 8 (10.8) | 13 (17.6) | 30 (40.5) | 10 (13.5) |
| 10. Conduct and Impulse Control Disorders | 43 (58.1) | 12 (16.2) | 23 (31.1) | 8 (10.8) | 7 (9.5) | 10 (13.5) | 41 (55.4) | 12 (16.2) |
| 11. Neuro-developmental Disorders | 30 (40.5) | 11 (14.9) | 16 (21.6) | 1 (1.4) | 3 (4.1) | 10 (13.5) | 47 (63.5) | 11 (14.9) |
| 12. Cognitive Disorders | 25 (33.8) | 9 (12.2) | 16 (21.6) | 3 (4.1) | 3 (4.1) | 10 (13.5) | 51 (68.9) | 5 (6.8) |
| 13. Obsessive-Compulsive Disorders | 68 (91.9) | 12 (16.2) | 26 (35.1) | 3 (4.1) | 4 (5.4) | 9 (12.2) | 28 (37.8) | 6 (8.1) |
| 14. Sleep Disorders | 67 (90.5) | 10 (13.5) | 23 (31.1) | 4 (5.4) | 6 (8.1) | 7 (9.5) | 27 (36.5) | 5 (6.8) |
| 15. Sexual Disorders | 53 (71.6) | 40 (54.1) | 20 (27.0) | 4 (5.4) | 5 (6.8) | 11 (14.9) | 34 (45.9) | 14 (18.9) |
| 16. Somatic Symptom Disorders | 57 (77.0) | 17 (23.0) | 26 (35.1) | 4 (5.4) | 6 (8.1) | 10 (13.5) | 37 (50.0) | 8 (10.8) |

ACT: Acceptance and Commitment Therapy; CBT: Cognitive Behavioural Therapy; DBT: Dialectical Behaviour Therapy;ECT: Electro-Convulsive Therapy; PTSD: Post-Traumatic Stress Disorder
