# Supplementary material for: A survey of perceived attitudes toward psychotherapy among Thai psychiatrists
Source: BMC Psychiatry. 2026 Jun 19;26:692. doi: 10.1186/s12888-026-08303-7 (PMC13540908; doi:10.1186/s12888-026-08303-7)
Supplement: Supplementary file 4 [file 12888_2026_8303_MOESM4_ESM.docx]

**Supplementary Table 4** Barriers to psychotherapy access. (N = 74)

| **Incentives for integrating psychotherapy ^a^** | **n (%)** |
| --- | --- |
| Economic factors (treatment will be more cost-effective) | 15 (20.3) |
| Immediate patient access to treatment | 2 (2.7) |
| Patient empowerment | 3 (4.1) |
| Clinical effectiveness of psychotherapy | 11 (14.9) |
| Patient adherence (easier to complete treatment, increased motivation) | 4 (5.4) |
| Patient attitudes (does not stigmatize, more convenient) | 3 (4.1) |
| Treatment capacity (health care system) | 35 (47.3) |
| Do not know | 0 (0.0) |
| Other (please write in the box) | 1 (1.4) |
| **Main barriers to integrating psychotherapy ^b^** |  |
| Economic/cost | 5 (6.8) |
| Degree of accessibility | 5 (6.8) |
| Psychotherapy is too time consuming | 34 (45.9) |
| Clinical effectiveness (decreased) | 1 (1.4) |
| Ethical (not a treatment that should be available) | 0 (0.0) |
| Professional/patient may not be skilled in psychotherapy | 7 (9.5) |
| Professional/patients’ attitudes (critical, non-believers, not ready for implementation) | 0 (0.0) |
| Systemic (mental health system is not ready for implementation) | 18 (24.3) |
| Stigma (treatment providers are against it, not interested) | 1 (1.4) |
| Patient safety (risks associated with sensitive patient information in treatment platforms) | 0 (0.0) |
| Cultural barriers (low level of education, taboo in revealing him/herself) | 1 (1.4) |
| Do not know | 1 (1.4) |
| Other (please write in the box) | 1 (1.4) |
| **Challenges for health care professionals ^c^** |  |
| Clinical effectiveness (low, psychotherapeutic method needs face-to-face interaction to work) | 5 (6.8) |
| Limited skills conducting psychotherapy | 8 (10.8) |
| Degree of accessibility and cost of access to treatment | 4 (5.4) |
| Use of psychotherapy is too time consuming | 30 (40.5) |
| Adherence, health care professional (low) | 2 (2.7) |
| Low motivation because of a critical attitude towards psychotherapy | 1 (1.4) |
| Not supported by health care system/health insurance | 6 (8.1) |
| Work overload | 18 (24.3) |
| Do not know | 0 (0.0) |
| Other (please write in the box) | (0.0) |

Note:
^a^ In your opinion what are the most important *incentives for integrating psychotherapy* as a treatment for mental disorders in your country?
^b^ What are the main barriers to integrating psychotherapy as a treatment for mental disorders?
^c^ What are the greatest *challenges for health care professionals* when providing psychotherapy for mental disorders?
