# Supplementary material for: A survey of perceived attitudes toward psychotherapy among Thai psychiatrists
Source: BMC Psychiatry. 2026 Jun 19;26:692. doi: 10.1186/s12888-026-08303-7 (PMC13540908; doi:10.1186/s12888-026-08303-7)
Supplement: Supplementary file 5 [file 12888_2026_8303_MOESM5_ESM.docx]

**Supplementary Table 5** Expected changes in psychotherapy training in Thailand: Type of professionals, platform and therapy formats

| **Expected changes in psychotherapy training** | **n (%)** | | | | | |
| --- | --- | --- | --- | --- | --- | --- |
| **Type of professionals ^a^** | **Not at all** | **Somewhat** | **Moderately** | **A lot** | **Very much** | **Not at all** |
| 1. Psychiatrists | 0 (0.0) | 3 (4.1) | 10 (13.5) | 15 (20.3) | 38 (51.4) | 8 (10.8) |
| 2. Psychologists | 0 (0.0) | 1 (1.4) | 2 (2.7) | 9 (12.2) | 56 (75.7) | 6 (8.1) |
| 3. Master-level counselors | 4 (5.4) | 4 (5.4) | 13 (17.6) | 19 (25.7) | 30 (40.5) | 4 (5.4) |
| 4. Clinical social workers | 2 (2.7) | 4 (5.4) | 23 (31.1) | 23 (31.1) | 19 (25.7) | 3 (4.1) |
| 5. Psychiatric nurses | 3 (4.1) | 5 (6.8) | 17 (23.0) | 31 (41.9) | 16 (21.6) | 2 (2.7) |
| 6. Personal coaches | 18 (24.3) | 19 (25.7) | 20 (27.0) | 9 (12.2) | 5 (6.8) | 3 (4.1) |
| 7. Master-level family therapists | 2 (2.7) | 1 (1.4) | 15 (20.3) | 21 (28.4) | 32 (43.2) | 3 (4.1) |
| 8. Self-help groups | 6 (8.1) | 6 (8.1) | 31 (41.9) | 15 (20.3) | 14 (18.9) | 2 (2.7) |
| 9. Peer counselors | 8 (10.8) | 9 (12.2) | 31 (41.9) | 17 (23.0) | 8 (10.8) | 1 (1.4) |
| 10. Paraprofessionals (e.g., people working alongside teachers to support students) | 7 (9.5) | 14 (18.9) | 28 (37.8) | 18 (24.3) | 6 (8.1) | 1 (1.4) |
| 11. Pastoral counselors (e.g., priests, imams, monks) | 5 (6.8) | 19 (25.7) | 26 (35.1) | 14 (18.9) | 6 (8.1) | 4 (5.4) |
| 12. Bachelor's degree-level therapists | 2 (2.7) | 11 (14.9) | 22 (29.7) | 16 (21.6) | 21 (28.4) | 2 (2.7) |
| **Type of platform: Therapy via ^b^** |  |  |  |  |  |  |
| 1. Videoconferencing | 4 (5.4) | 5 (6.8) | 16 (21.6) | 17 (23.0) | 32 (43.2) | 0 (0.0) |
| 2. Multiple or flexible platforms | 1 (1.4) | 6 (8.1) | 17 (23.0) | 18 (24.3) | 32 (43.2) | 0 (0.0) |
| 3. Smartphone apps | 4 (5.4) | 8 (10.8) | 20 (27.0) | 14 (18.9) | 28 (37.8) | 0 (0.0) |
| 4. Telephone | 12 (16.2) | 12 (16.2) | 26 (35.1) | 6 (8.1) | 18 (24.3) | 0 (0.0) |
| 5. Texting | 18 (24.3) | 16 (21.6) | 24 (32.4) | 6 (8.1) | 10 (13.5) | 0 (0.0) |
| **Therapy formats ^c^** |  |  |  |  |  |  |
| 1. Psychoeducational groups for specific issue | 2 (2.7) | 8 (10.8) | 14 (18.9) | 16 (21.6) | 26 (35.1) | 8 (10.8) |
| 2. Individual therapy | 4 (5.4) | 6 (8.1) | 12 (16.2) | 14 (18.9) | 31 (41.9) | 7 (9.5) |
| 3. Crisis intervention | 3 (4.1) | 8 (10.8) | 12 (16.2) | 14 (18.9) | 29 (39.2) | 8 (10.8) |
| 4. Very short-term therapy (1-3 sessions) | 3 (4.1) | 5 (6.8) | 18 (24.3) | 14 (18.9) | 27 (36.5) | 7 (9.5) |
| 5. Short-term therapy (5-12 sessions) | 2 (2.7) | 7 (9.5) | 14 (18.9) | 22 (29.7) | 22 (29.7) | 7 (9.5) |
| 6. Long-term therapy | 4 (5.4) | 14 (18.9) | 16 (21.6) | 14 (18.9) | 19 (25.7) | 7 (9.5) |
| 7. Couple/Marital therapy | 2 (2.7) | 9 (12.2) | 14 (18.9) | 13 (17.6) | 28 (37.8) | 8 (10.8) |
| 8. Group therapy | 2 (2.7) | 10 (13.5) | 18 (24.3) | 9 (12.2) | 27 (36.5) | 8 (10.8) |
| 9. Family therapy | 2 (2.7) | 7 (9.5) | 18 (24.3) | 13 (17.6) | 26 (35.1) | 8 (10.8) |
| 10. Population-level interventions (Policies/programs) | 3 (4.1) | 10 (13.5) | 17 (23.0) | 13 (17.6) | 20 (27.0) | 11 (14.9) |

Note:
^a^ How likely are you to *expect other professionals to provide psychotherapy to your patients?*
^b^ How likely are you to expect changes in the delivery of psychotherapy?
^c^ How likely are you to *expect changing psychotherapy structure*?
