# Supplementary material for: A survey of perceived attitudes toward psychotherapy among Thai psychiatrists
Source: BMC Psychiatry. 2026 Jun 19;26:692. doi: 10.1186/s12888-026-08303-7 (PMC13540908; doi:10.1186/s12888-026-08303-7)
Supplement: Supplementary file 6 [file 12888_2026_8303_MOESM6_ESM.docx]

**Supplementary Table 6** Internet-based psychotherapy: To what extent are you familiar with *internet-based psychotherapy* (also referred to as online therapy and web-based treatment)? (N = 74)

| **Familiarity with internet-based psychotherapy** | **n (%)** |
| --- | --- |
| 1 = “no knowledge at all” | 14 (18.9) |
| 2 = “some knowledge” | 18 (24.3) |
| 3 = “moderate knowledge” | 16 (21.6) |
| 4 = “good knowledge” | 14 (18.9) |
| 5 = “very good knowledge” | 7 (9.5) |
| Does not apply | (6.8) |
