# Supplementary material for: A survey of perceived attitudes toward psychotherapy among Thai psychiatrists
Source: BMC Psychiatry. 2026 Jun 19;26:692. doi: 10.1186/s12888-026-08303-7 (PMC13540908; doi:10.1186/s12888-026-08303-7)
Supplement: Supplementary file 7 [file 12888_2026_8303_MOESM7_ESM.docx]

**Supplementary Table 7** Internet-based psychotherapy: In your opinion, what are some of the *challenges* *for patient* receiving internet-based psychotherapy? (N = 74)

| **Challenges for patient receiving internet-based psychotherapy** | **n (%)** |
| --- | --- |
| Clinical effectiveness (low, psychotherapeutic method needs face-to-face interaction to work) | 33 (44.6) |
| Difficulties with applying self-help strategies | 17 (23.0) |
| Degree of accessibility and cost of access to treatment | 24 (32.4) |
| Use of psychotherapy is too time consuming | 13 (17.6) |
| Patient adherence (low) | 21 (28.4) |
| Low motivation because of a critical attitude towards psychotherapy | 13 (17.6) |
| Not supported by health care system/health insurance | 25 (33.8) |
| Patient attitude (stigma/taboo in revealing him/herself etc. that generate more difficulty in the use of psychotherapy) | 5 (6.8) |
| Do not know | 2 (2.7) |
| Other (please write in the box) | 6 (8.1) |
